# IgA expressed by glomerular mesangial cells is involved in the pathogenesis of IgA nephropathy

**DOI:** 10.3389/fimmu.2025.1638818

**Published:** 2025-11-25

**Authors:** Haidong Zhang, Zhenling Deng, Yueming Gao, Qi Li, Lu Zhang, Yue Wang

**Affiliations:** Department of Nephrology, Peking University Third Hospital, Beijing, China

**Keywords:** IgA nephropathy, non-B immunoglobulin, galactose-deficient IgA1, μMT mice, GMC conditional *IGHA* knockout mice

## Abstract

**Background:**

The widely accepted “multi-hit hypothesis” of IgAN pathogenesis was challenged, as efficient depletion of CD20+ B cells failed to reduce serum galactose-deficient IgA1 (Gd-IgA1) or proteinuria in IgAN patients. Our group has discovered glomerular mesangial cells (GMCs) as another source of IgA, while immunoglobulin produced by non-B cells (non-B Ig) participating in several inflammatory and neoplastic diseases arose as a new concept in immunology. It is still unclear whether IgA produced by GMCs participates in the pathogenesis of IgAN and what its preliminary mechanism is.

**Methods:**

The transcription of *IGHA1* and its associated function in GMCs were demonstrated by single-cell RNA sequencing (scRNA-seq) analysis. *IGHA1* transcription in glomerular mesangium was detected in para-cancerous renal tissues by fluorescence *in situ* hybridization (FISH). *Staphylococcus aureus* enterotoxin B (SEB), Toll-like receptor 4 (TLR4) antagonist, and small interfering RNA (siRNA) were used to investigate the pro-inflammatory effect of Gd-IgA1 and its overproduction pathway. The IgAN model was established in μMT mice (lacking B lymphocytes with reduced serum IgA) and mice with *IGHA* conditional knockout in GMCs to observe the causality between GMC-expressed IgA and the formation of IgAN.

**Results:**

Expression of IgA in GMCs was reconfirmed by detecting *IGHA1* transcription in single cells and in para-cancerous renal tissue *in situ*. Gene set enrichment analysis (GSEA) indicated that *IGHA1* transcription in GMC was significantly associated with response to bacterium, innate immune response, complement activation, and galactose metabolism. Cultured human GMC experiments revealed that SEB could stimulate Gd-IgA1 overproduction through the TLR4 signaling pathway, and Gd-IgA1 deficiency in GMCs relieved the extracellular matrix component (ECM) deposition and C3 and IL-6 production induced by SEB. Mesangial IgA deposition and ECM expansion pattern in the μMT mouse IgAN model were similar to those in Balb/c mice, and mice with *IGHA* conditional knockout in GMCs relieved glomerular inflammatory response and alleviated the hematuria and proteinuria in the mouse IgAN model.

**Conclusion:**

We reconfirmed the expression of IgA in GMCs and demonstrated that overexpression of Gd-IgA1 in GMCs induced by SEB through the TLR4 pathway in human GMC may play an important role in inducing an inflammatory response in IgAN.

## Introduction

IgA nephropathy (IgAN) is the most common primary glomerular disease and the leading cause of kidney failure in the world (1), with approximately 30% to 40% patients ending in ESRD within 10 to 20 years after diagnosis (2). No specific and causal treatment has been developed for IgAN since the first report of an IgAN patient by Berger (3) due to stagnant understanding of the pathogenesis of IgAN, particularly the mechanism by which nephritogenic IgA is deposited specifically in the glomerular mesangium (4, 5).

The clinical presentation of IgAN is asymptomatic hematuria with varying degrees of proteinuria and kidney insufficiency, and its pathological lesion is deposition of dominant galactose-deficient IgA1 (Gd-IgA1) in the mesangium with mesangial cell proliferation and extracellular matrix (ECM) expansion (6). The widely accepted “multi-hit hypothesis” proposes that Gd-IgA1 produced by B cells in gut mucosa and plasma cells in bone marrow (7, 8) served as a self-antigen and triggered the generation of IgG autoantibodies, forming the circulating Gd-IgA1–IgG immune complex to deposit in the glomerular mesangium and initiate an inflammation response of GMC. However, it brought a challenge to this hypothesis that rituximab efficiently depleted circulating CD20^+^ B cells but failed to reduce the serum Gd-IgA1, autoantibody IgG, and proteinuria in IgAN patients (9), and it is difficult to clarify the mechanism by which large molecular size Gd-IgA1–IgG immune complex from peripheral blood could penetrate the endothelium and deposit in the mesangium (10).

The classical theory holds that B lymphocytes and plasma cells were the only source of immunoglobulin (known as B-Ig), while several studies confirmed that almost all tumor cells (11), normal tissue cells (12–14), and immune-privileged cells (15, 16) can express IgA (called non-B-Ig). Compared with B-Igs, the variable region of non-B-Igs was relatively conservative with strong hydrophobicity, which leads them to accumulate or aggregate. Physiologically, non-B-Igs participate in local immunity, maintenance of cell proliferation, and formation of cytoskeletal proteins (11–14), while non-B-Igs induced by pathogenic factors participate in immune and inflammatory diseases such as hepatitis (12) and inflammatory bowel disease (13). Gd-IgA1 deposited in the glomerular mesangium in IgAN presents features of non-B-Igs, suggesting that the locally deposited Gd-IgA1 might come from non-B cells.

Our research team previously found that renal intrinsic cells including GMCs (17, 18), glomerular podocytes (19, 20), and proximal tubular epithelial cells (21, 22) could express various kinds of non-B-Igs, and GMCs could express IgA (dominantly IgA1) functioning in proliferation and migration. Additionally, Liu (23, 24) reported the transcription of *IGHA* in mouse GMCs. These studies suggested that GMCs producing IgA might contribute to IgA deposition in the mesangium in IgAN.

In this present study, we reconfirmed the expression of IgA and Gd-IgA1 in GMCs and found that overexpressed Gd-IgA1 in GMCs induced by SEB through the TLR4 pathway stimulated the ECM deposition and C3 and IL-6 production. Increased IgA deposition with excessive ECM deposition in glomerular mesangium could be induced in a B-cell-deficient condition in μMT mice, similar to Balb/c mice, and *IGHA* conditional knockout in GMC relieved glomerular inflammatory response and alleviated the hematuria and proteinuria in the mouse IgAN model.

## Materials and methods

### Cell culture

The culture protocol of the primary human GMC (HRMC) and the human GMC line (C2M12) was described in detail in our previous study (17).

### Cell stimulation

HRMC and C2M12 were seeded uniformly on six-well plates. When the subcultured cells reached 70% confluency, GMCs were cultured in a medium containing 0.5% FBS overnight for synchronization, followed by treatment with 1 μg/mL of SEB (Nanjing Aiyi Biotechnology Co., Ltd., Nanjing, China), *Staphylococcus aureus* Cowan 1 (SAC) (1:1,000), or lipopolysaccharide (LPS) (5 μg/mL) in a serum-free culture medium for 48 h.

### Transfection in cultured C2M12 with small interfering RNA

Lipofectamine 3000 (Invitrogen; Thermo Fisher Scientific, Inc. Massachusetts, USA) was used for the transfection with small interfering RNA (siRNA). siRNAs directed against different regions of the constant region of the *IGHA1* (siRNA-1 and siRNA-2) and the non-specific, scrambled, control siRNA (NC) were designed by GenePharma Company (Shanghai, China) (**Supplementary Table S1**). The C2M12 was transfected with siRNA in a serum-free culture medium for 48 h following the Lipofectamine 3000 protocol.

### Reverse transcription-quantitative polymerase chain reaction and PCR

Total RNA of cultured cells was extracted with the RaPure Total RNA Kit (Magen, Inc., Guangzhou, China), and its concentration was assessed using a NanoDrop spectrophotometer (Thermo Fisher Scientific, Inc.). Then, 2 μg of total RNA was reverse-transcribed to cDNA using the RevertAid First Strand cDNA Synthesis Kit (Thermo Fisher Scientific, Inc.). The FastStart Universal SYBR Green Master Mix kit (Roche, Mannheim, Germany) was used for quantitative polymerase chain reaction (qPCR), with the primers targeting complement C3 (C3), interleukin 6 (IL-6), collagen type IV alpha 1 (COLIVA1), fibronectin 1 (FN1), and the constant region of immunoglobulin heavy constant alpha 1 (*IGHA1*). The reaction conditions regulated by the 7500 FAST real‐time PCR system (Applied Biosystems, USA) and the reaction conditions for standard PCR are listed in **Supplementary Table S2**. HRMC cDNA was also used to amplify the hinge region of *IGHA1* covering the serine/threonine-rich region by PCR with primers targeting the CH1-H-CH2 region. The sequences of the primers are listed in **Supplementary Table S3**. PCR products of the *IGHA1* CH1-H-CH2 region amplification were sent to BGI.Tech (Beijing, China) for sequencing, and the sequence was analyzed by the Chromas software.

### Western blot analysis

The cell lysis solution, extracellular matrix component (ECM), and culture supernatant were harvested for Western blot analysis. Cultured cells were detached from the bottom of the six-well plate by incubating with 1 mL of Versene solution (NaCl 8 g/L, KCl 0.2 g/L, Na_2_HPO_4_*12H_2_O 2.3 g/L, KH_2_PO_4_ 0.2 g/L, EDTANa_2_ 0.2 g/L, pH 7.2–7.4) in each well (37°C, 10 min) and collected into 15 mL centrifuge tubes. Thus, the ECM remained intact and deposited at the bottom of the plate. The cell pellet was obtained after centrifugation for cell lysis. An isochoric TSD lysis buffer (TSD lysis buffer, 1% SDS; 50 mmol/L, pH 7.5 Tris–HCl) was added into each well of the six-well plates to collect the ECM. The culture supernatant was concentrated by the ultrafiltration centrifuge tube (Merck, Darmstadt, Germany) into an isochoric solution. The samples after denaturation were immediately used for Western blot analysis in non-reduced and reduced conditions. Serum from IgAN patients and the culture medium were used as positive control and negative control, respectively.

### Human subjects

Renal biopsy specimens from IgAN patients and para-cancerous renal cortices from patients with renal carcinoma were obtained with informed consent from patients and approval of the Research Ethics Committee, Peking University Third Hospital, Beijing.

The inclusion criteria for IgAN patients were listed as follows: 1) pathologically confirmed primary IgAN, 2) 18–45 years old, 3) eGFR >30 mL/min/1.73 m^2^, and 4) urinary protein <3.5 g/day. The exclusion criteria for IgAN patients were as follows: 1) secondary IgAN such as disease associated with viral infections, autoimmune diseases, hepatic cirrhosis, and tumors; 2) IgAN accompanying other renal diseases such as crescentic nephritis, minimal change disease, membranous nephropathy or other nephropathy, and transplanted kidney IgAN; 3) use of immunosuppressive agents; and 4) renal replacement therapy has been performed.

The inclusion criteria for para-cancerous renal cortices from patients with renal carcinoma included the following: 1) the renal cortices were obtained far enough from the tumor boundary (at least 5 cm away) to prevent transcription alteration due to malignancy differentiation and 2) patients without hypertension, diabetes, hepatitis B, tuberculosis, amyloidosis, systemic lupus erythematosus vasculitis, or Sjogren’s syndrome.

### Identification of glomerular mesangial cells from human para-cancerous renal cortices and detection of *IGHA* gene rearrangement and transcription

Renal tissues were mixed and minced into small pieces (<1 mm^3^ in size) in ice-cold HBSS^−/−^ using a razor blade. These small pieces were passed through a 100-μm cell strainer, the flow-through was then passed through a 70-μm cell strainer, and the retained glomeruli were transferred onto the slides for isolation. Single glomeruli were isolated under a microscope by capillary pipette and digested in PBS containing 1 mg/mL of collagenase I (Sigma Chemicals, St. Louis, MO, USA) for 5 min at 37°C. Glomerular intrinsic cells were released, and single glomerular cells were manually picked out under the microscope by a capillary pipette.

RNA extraction, cDNA synthesis, and amplification of these single glomerular cells were carried out following the protocol we previously described (19). *ITGA8* and *ACTA2* were considered as mesangial cell-specific markers (25). The sequences of the primers are listed in **Supplementary Table S3**. The specific V_H_DJ_H_ rearrangement sequence of *IGHA* mRNA in these single GMCs was amplified by nested PCR and compared with those in the basic local alignment search tool (IgBLAST, https://www.ncbi.nlm.nih.gov/igblast/) to identify the best-matching germline gene segments and junctions. Detailed protocols including reaction conditions and primers used for nested PCR were described in the previous study by our team (19).

### Reanalysis of the kidney single-cell RNA sequencing data from the public database

Kidney single-cell RNA sequencing (scRNA-seq) data were downloaded from the public scRNA-seq database (DISCO, DISCO (immunesinglecell.org). The data processing, quality control, and filtering were conducted in R using the Seurat package (Version 4.3.0). Each *IGHA1*-expressing cell cluster was assigned to a corresponding cell type based on known cell-type markers. Mesangial cell-type markers include *PDGFRB*, *ITGA8*, *LUM*, and *COL1A2* (25), and *CD19*, *CD27*, and *CD38* were considered specific markers for B and plasma cells.

Gene set enrichment analysis was performed to explore the *IGHA1*-associated biological functions and pathways in *IGHA1*-expressing GMCs, in which the c2.cp.kegg_legacy.v2024.1.Hs.symbols.gmt, c2.cp.reactome.v2024.1.Hs.symbols.gmt, and c5.go.bp.v2024.1.Hs.symbols.gmt were used as reference genomes. An adjusted *P-*value <0.05 acquired through the Benjamini–Hochberg approach was considered statistically significant.

### Mice

*FOXD1*^GC^ mice and *IGHA*^flox^ (*IGHA*^fl^) mice were purchased from the Jackson Lab and the Biocytogen Pharmaceuticals (Beijing), respectively. *IGHA* homozygous and Foxd1 heterozygous mice were bred to generate *FOXD1*^GC^*IGHA*^fl/fl^ mice. As Foxd1 is mainly expressed in GMCs, we considered *FOXD1*^GC^*IGHA*^fl/fl^ mice as the GMC conditional *IGHA* knockout mice (cKO mice), and the *IGHA* homozygous mice (*IGHA*^fl/fl^) without the Foxd1 cre allele, named as wild-type mice (WT mice), were used as littermate controls. The PCR conditions and primers used for mice genotyping are listed in **Supplementary Tables S2, S3**.

Ighmtm1Cgn (μMT) mice, which lack serum IgA and B cells (26), were a kind gift from Professor Qiu (Peking University Health Science Center, Beijing, China). Balb/c mice were purchased from the Charles River Laboratories (Beijing, China). Animal experimentation was performed following the NIH Guide for the Care and Use of Laboratory Animals.

### Establishment of the IgAN mouse model

Male mice aged 8–10 weeks were utilized for the experiment. In the IgAN model group, each mouse was given 0.4 mL of 6 mmol/L hydrochloric acid solution containing 0.1% BSA (1 g/L) (Sigma-Aldrich, Inc., USA) every 2 days via oral gavage in the first 5 weeks, followed by tail intravenous injection of 0.2 mL of 0.01 mol/L PBS containing 2% BSA for three consecutive days in the sixth week. From the 9th to the 11th week, 0.1 mL of SEB solution (diluted in PBS) was administered to each mouse (0.5 mg/kg) via tail intravenous injection once a week. In the control group, 0.4 mL of 6 mmol/L hydrochloric acid solution was given in the first 5 weeks, and PBS was intravenously injected on the 6th and 9th to 11th weeks.

Mice were sacrificed on the 12th week, and 24-h urine was collected in the metabolic cage 1 day before the harvest. Hematuria, calculated by red blood cell count per microliter in urine, was measured by flow cytometry, and urine albumin–creatinine ratio (UACR) and serum urea and serum creatinine levels were measured using a biochemical analyzer, respectively, in the clinical laboratory in our hospital. Cardiac perfusion with PBS was performed to remove the intrarenal blood before kidney harvest.

### Histological analyses

RNA-fluorescence *in situ* hybridization (FISH) was performed on human para-cancerous kidney tissue using the RNASweAMI fluorescence *in situ* hybridization detection kit (GF007; Servicebio, Hubei, China), and the FISH probe targeting human *IGHA1* was designed and synthesized by Servicebio. After hybridization following the recommended protocol, we co-stained the Pdgfrb protein to display glomeruli at 37°C for 1 h, followed by a 1-h secondary antibody incubation at room temperature.

For indirect immunofluorescence (IF) staining, cells cultured on confocal dishes were fixed and permeabilized with 4% paraformaldehyde and 0.1% Triton X-100. Mice kidney samples embedded in OCT were fixed and permeabilized by cold acetone. Fluorescent signals were detected with a confocal laser scanning microscope (Carl Zeiss, Germany), and the intraglomerular mean fluorescence intensity was quantitatively measured by ZEN 2012 SP2.

Immunohistochemistry (IHC) staining was performed on the formalin-fixed paraffin-embedded kidney tissues. We incubated the sections with 10 mM of citrate buffer (pH = 6.0) in a pressure cooker for 3 min for antigen retrieval. The intraglomerular mean optical density (mOD) of the specific antibody staining was quantitatively analyzed by the IHC Toolbox plugin in the ImageJ software.

Masson’s trichome staining and hematoxylin and eosin (HE) staining were performed following the protocols in the Masson Trichrome Staining Kit and the HE Stain Kit (Solarbio, Beijing, China), respectively. At least 10 glomeruli with maximal cross section for each mouse were randomly chosen for histological statistical analysis.

### Antibodies

The following antibodies were used: Gd-IgA1 (1:1,000 in WB, 1:20 in IF; KM55; Immuno-Biological Laboratories, Gunfecture, Japan), IgA (1:1,000 in WB, 1:200 in IF; ab124716; Abcam, Cambridge, UK), COLIV (1:1,000 in WB, 1:2,000 in IHC; ab236640; Abcam), FN (1:1,000 in WB, 1:2,000 in IHC; ab268022; Abcam), PDGFRB (1:100; ab32570; Abcam), C3 (1:1,000 in WB, 1:2,000 in IHC; ab200999; Abcam), IL-6 (1:1,000; ab290735; Abcam), and C1GALT1 (1:3,000 in WB, 1:300 in IF; ab237734; Abcam).

Aside from using the anti-IgA (1:20; Gene Tech Company Limited, Shanghai, China) antibody for direct IF was officially used for the detection of glomerular IgA deposition in IgAN diagnosis in the Pathology Department at Peking University Third Hospital.

### Statistical analysis

At least three independent biological repeats were performed in every experiment. The significant differences between groups were calculated by Student’s unpaired *t*-test and one-way ANOVA with Tukey’s multiple comparison test. A *P*-value <0.05 was considered statistically significant. All statistical analyses were performed using GraphPad Prism version 8.0 for Windows (GraphPad Software, San Diego, CA).

## Results

### *IGHA* gene transcription in single GMCs

**Figure 1:**
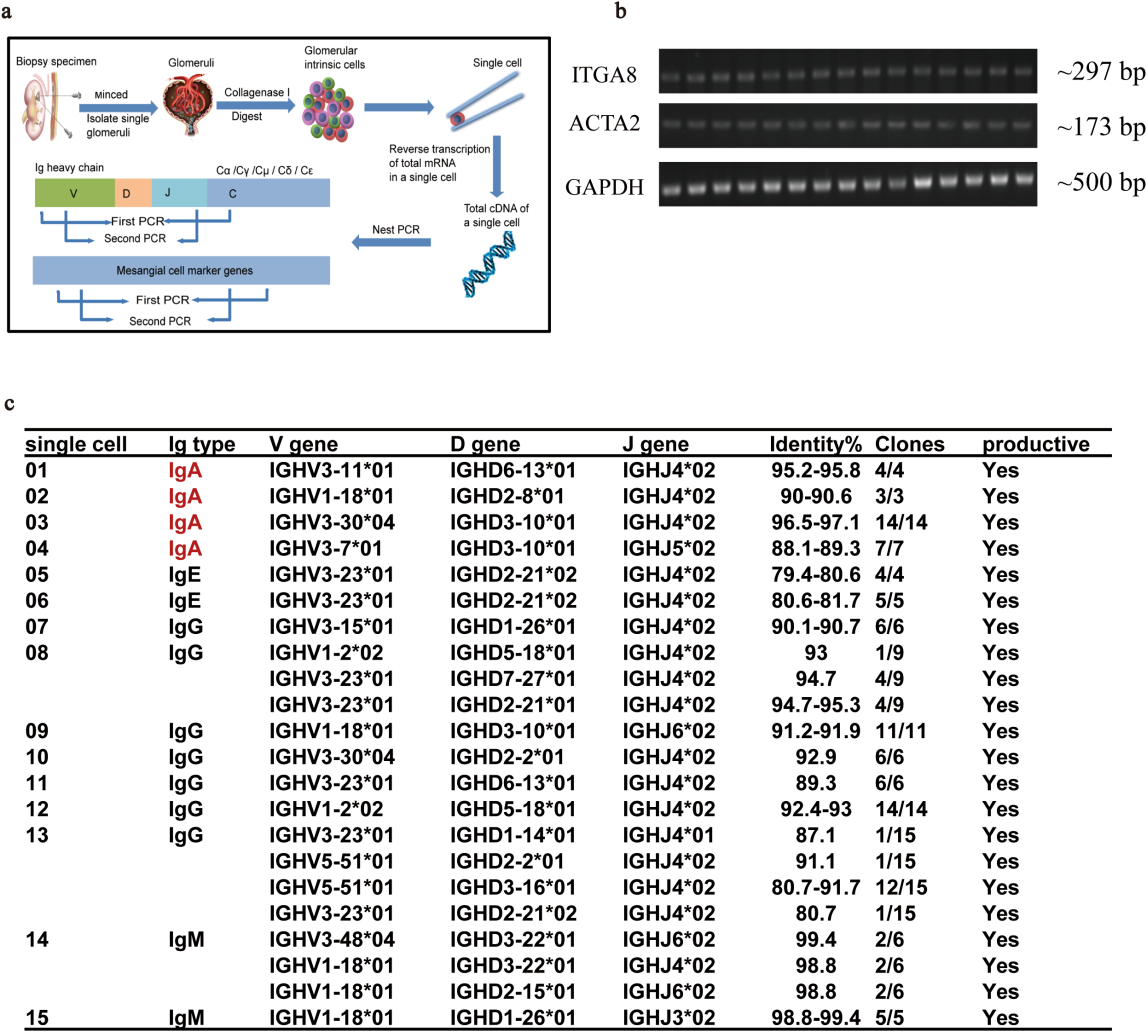
Nested PCR combined with Sanger sequencing confirmed the transcription of the variable region of *IGHA* in a single GMC isolated from para-cancerous renal cortices. **(a)** The procedure for single GMC isolation and the identification of the V_H_DJ_H_ sequence of *IGHA*. **(b)** Transcription feature of the 15 isolated GMCs. **(c)** The matched V_H_DJ_H_ rearrangement patterns of different immunoglobulin types in single GMCs. PCR, polymerase chain reaction; *IGHA*, immunoglobulin heavy constant alpha; GMC, glomerular mesangial cell; IgAN, immunoglobulin A nephropathy.

Nested PCR on individual glomerular cells was used to demonstrate the expression of *IGHA* in isolated GMCs. Single glomerular cells were isolated from para-cancerous renal cortices to detect *IGHA* V_H_DJ_H_ gene rearrangement and transcription (**Figure 1a**). Fifteen single cells with transcription features of *ITGA8* and *ACTA2* were identified as GMCs (**Figure 1b**), of which four GMCs were detected to express the variable region of the *IGHA* transcripts. Transcription of the variable region of *IgHE*, *IgHG*, and *IgHM* was also detected in other GMCs (**Figure 1c**).

**Figure 2:**
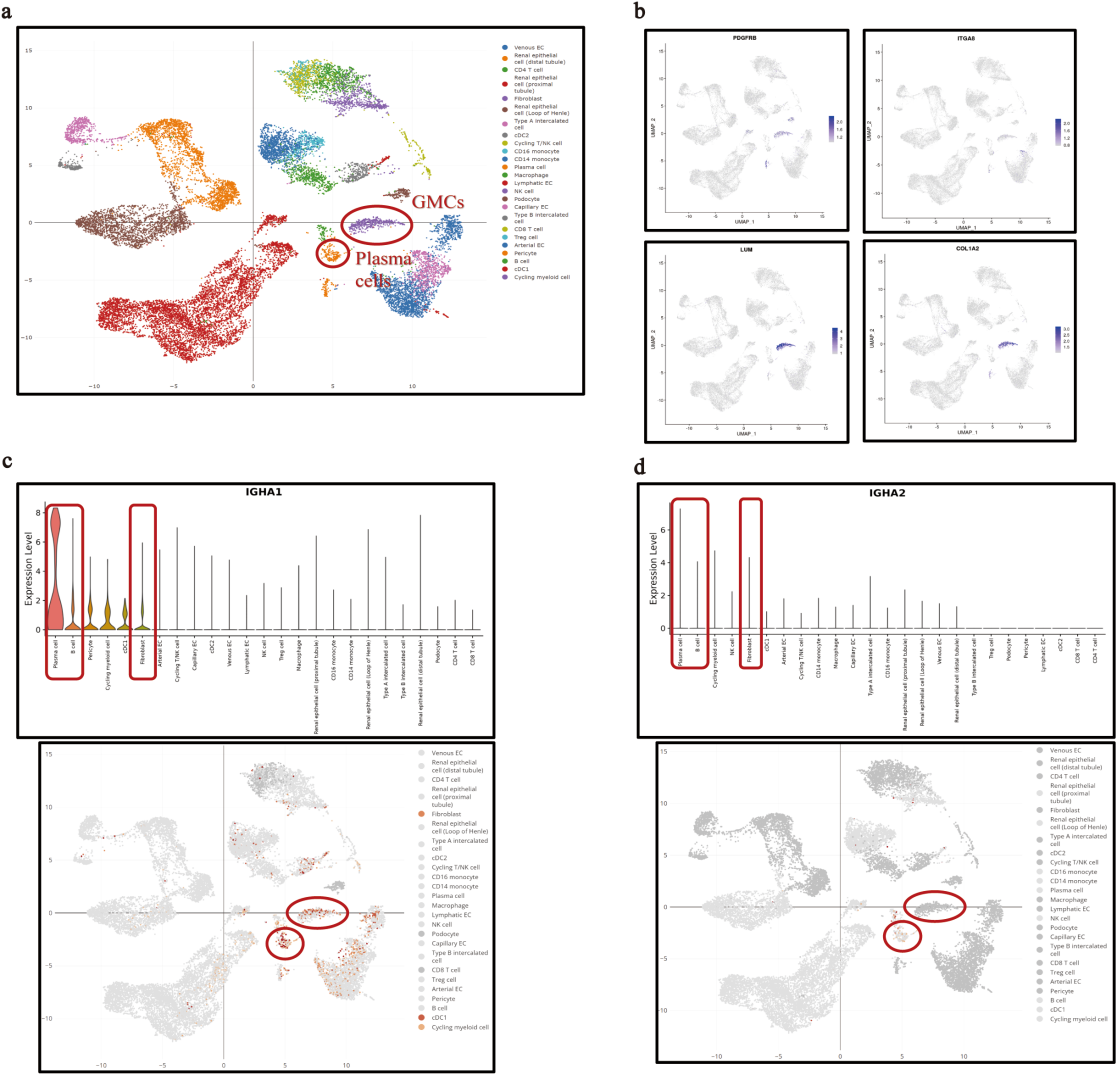
*IGHA* transcription in single GMCs in the public single-cell RNA sequencing database. **(a)** Interactive UMAP illustration of integrated scRNA-seq data of kidney cells colored by cell-type annotation in the database. **(b)** Identification of the fibroblasts annotated in the database as glomerular mesangial cells with specific transcription features. **(c, d)** Transcription of *IGHA1***(c)** and *IGHA*2 **(d)** in plasma cells, B cells, and GMCs. *IGHA*, immunoglobulin heavy constant alpha; GMC, glomerular mesangial cell; RNA, ribonucleic acid; UMAP, uniform manifold approximation and projection; scRNA-seq, single-cell RNA sequencing.

The transcription of *IGHA1* in GMCs was reconfirmed in the public single-cell RNA-sequencing database (DISCO, DISCO (immunesinglecell.org), in which fibroblasts with transcription features of *PDGFRB+ITGA8+LUM+COLIA2+* were considered as GMCs (**Figures 2a, b**). It was reported that GMCs present with fibroblast features (27). This group of cells was not B cells or plasma cells, as they did not express *CD19*, *CD27*, or *CD38* (**Supplementary Figure S1a**). Although the *IGHA* transcriptional level in GMCs was relatively lower compared with that in plasma cells, *IGHA1* was the dominant one in both cells (**Figures 2c, d**). Reclustering of the *IGHA1*-expressing cells in the database based on the known cell-type markers additionally revealed that not only immune cells but also renal parenchymal cells including mesangial cells, podocytes, tubular epithelial cells, pericytes, and endothelial cells could express *IGHA1* (**Supplementary Figure S1b**). Further investigation of the differential expression pattern among these *IGHA1*-expressing cells could prevent these *IGHA1*-expressing renal parenchymal cells from B-cell or plasma-cell contamination. **Supplementary Figure S1c** lists the top 8 genes differentially expressed in each *IGHA1*-expressing cell type.

### *In situ* IgA expression in para-cancerous renal tissue and in μMT mice

Following our finding that expression of *IGHA* was identified in single GMCs, we detected IgA expression in glomerular mesangium *in situ* in human para-cancerous renal tissue and in μMT mice.

**Figure 3:**
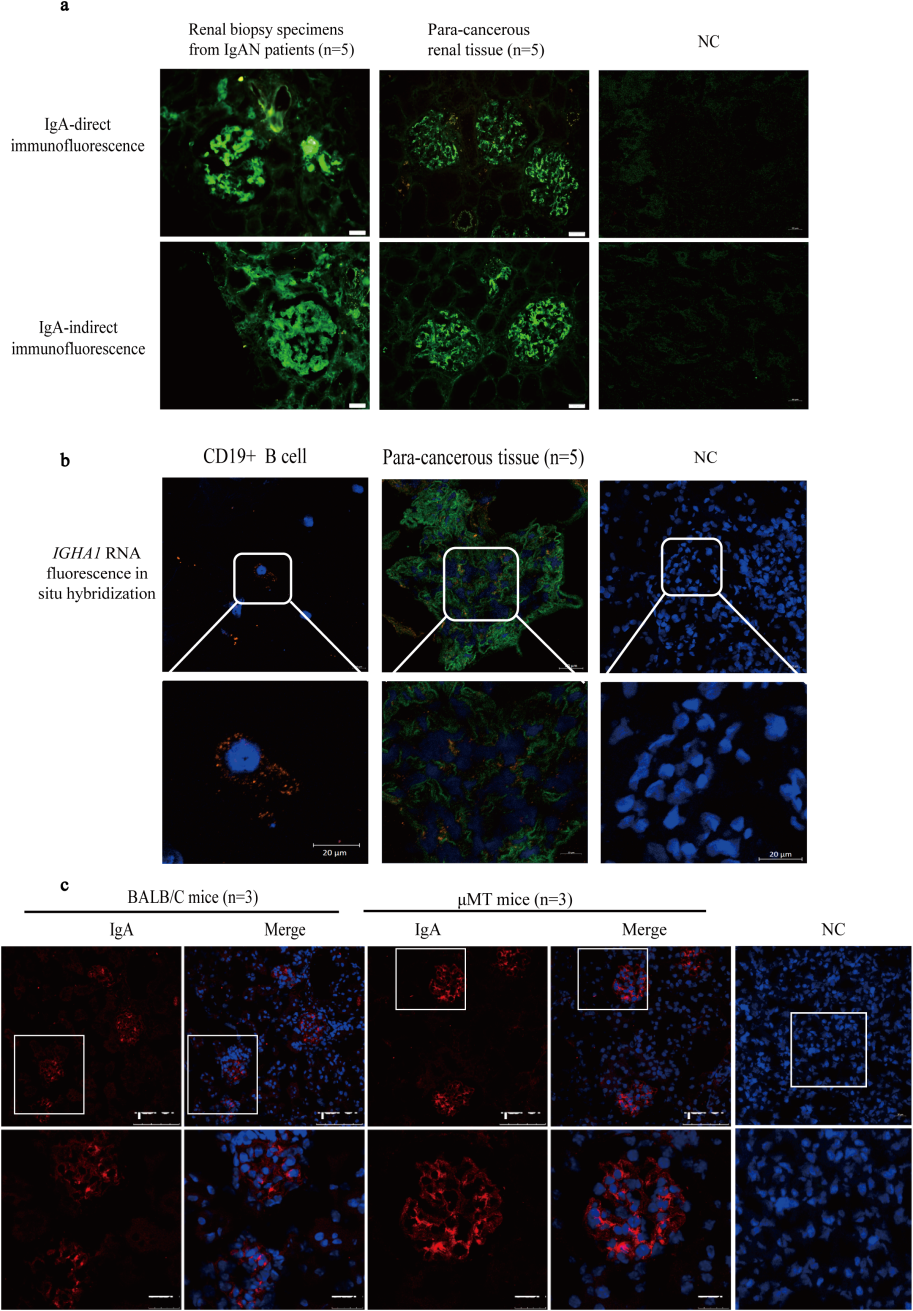
The IgA expression was observed in normal renal tissue and μMT mice in addition to IgAN patients. **(a)** The IgA staining in glomerular mesangium in IgAN patients and in para-cancerous renal tissues from patients with renal carcinoma. **(b)***IGHA1* RNA detection in CD19^+^ B cells and glomeruli in para-cancerous kidney tissue by FISH (orange) containing the GMC marker Pdgfrb by immunofluorescence (green). **(c)** The IgA staining in Balb/c and μMT mice. IgA, immunoglobulin A; μMT, Ighmtm1Cgn; IgAN, immunoglobulin A nephropathy; NC, negative control; FISH, fluorescence *in situ* hybridization; GMC, glomerular mesangial cell; Pdgfrb, platelet-derived growth factor receptor beta.

IgA protein deposition in glomerular mesangium in para-cancerous renal tissues was detected clearly although IgA staining was light and sparse compared to the thick and dense staining in IgAN renal biopsies (**Figure 3a**). More importantly, fluorescent hybridization of *IGHA1* RNA (orange) and Pdgfrb protein co-staining (green) reconfirmed the *IGHA1* transcription by GMCs *in situ* (**Figure 3b**). To exclude the presence of IgA from local B lymphocytes or the infiltrated serum IgA, IgA expression in glomerular mesangium was detected in μMT mice lacking B lymphocytes and serum IgA (28), and it was usually used to study the effect of B-lymphocyte-originated immunoglobulin (29). The IgA expression in μMT mice was clear and precise, even with a higher intensity than that in wild-type mice (possibly due to the weakened inhibitory effect of systemic immunity on local immunity (30)) (**Figure 3c**).

### Gd-IgA1 expression in human GMCs

Gd-IgA1 deposition in glomerular mesangium is the most important factor that initiates the pathogenesis of IgAN. A serine/threonine-rich region was detected in the *IGHA1* transcripts in HRMC (**Supplementary Figure S2**). While HRMC transcribed the CH1-H-CH2 sequence in the *IGHA1* constant region with a similar length to PBMC (**Supplementary Figures S2a, b**), the amino acid composition of its serine/threonine-rich region (containing 6 serine/threonine in 18 amino acids) is different from that in B lymphocytes (**Supplementary Figure S2c**) (31). The presence of a serine/threonine-rich region in *IGHA1* transcripts provided the possibility of galactose deficiency in IgA1 produced by cultured GMCs.

**Figure 4:**
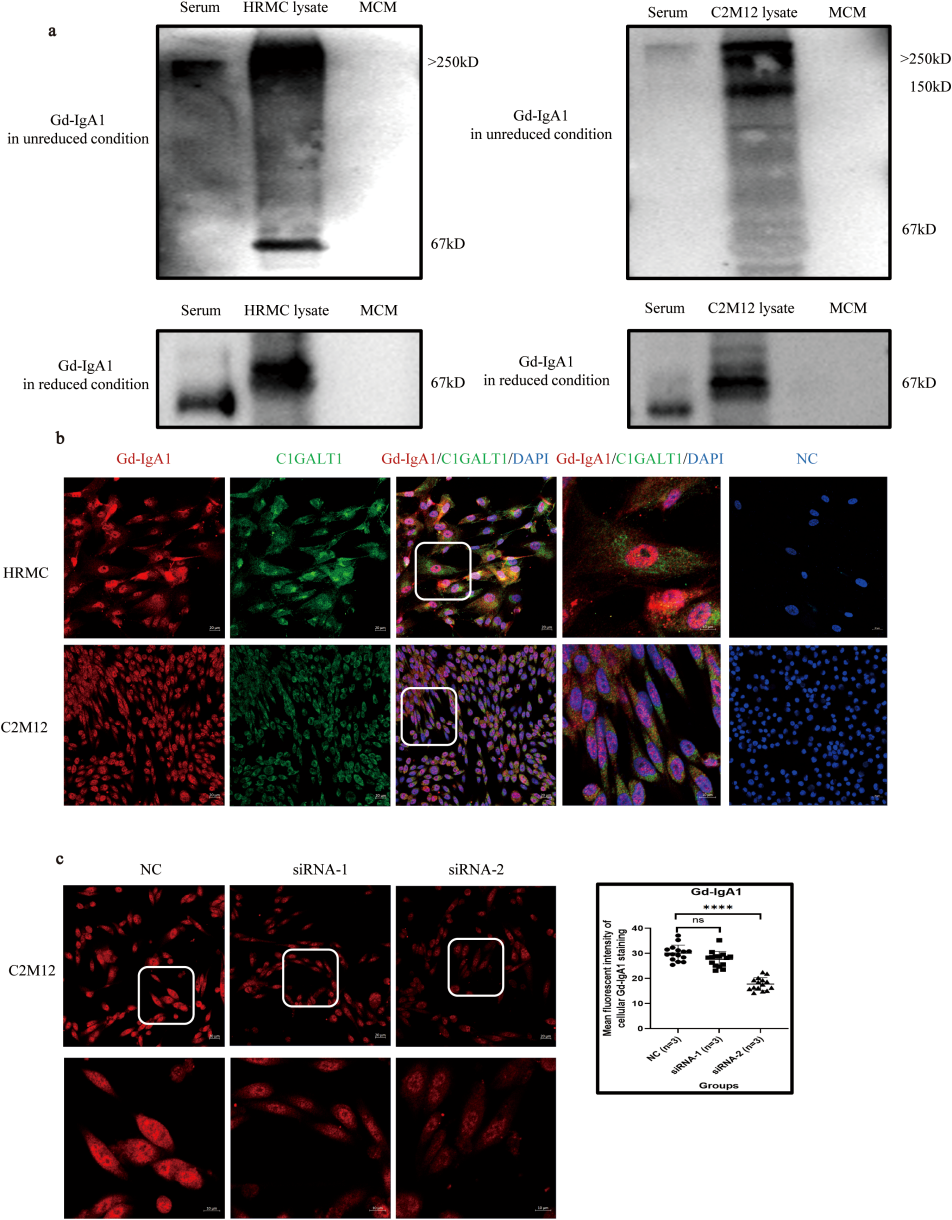
Expression of Gd-IgA1 in HRMC and C2M12. **(a)** Western blot analysis of Gd-IgA1 expression in HRMC and C2M12 in unreduced and reduced conditions. **(b)** Immunofluorescence of Gd-IgA1 and C1GALT1 expression in HRMC and C2M12. **(c)** Immunofluorescence of Gd-IgA1 staining in C2M12 after siRNA transfection (*n* = 3 per group; ****P < 0.0001). HRMC, human renal mesangial cell; Gd-IgA1, galactose-deficient immunoglobulin A1; C1GALT1, core 1β1,3-galactosyltransferase; siRNA, small interfering RNA; NC, negative control; ns, not significant.

Western blot demonstrated that Gd-IgA1 in unreduced condition in both HRMC and C2M12 cytoplasm was larger than 250 kDa, similar to that in the serum from IgAN patients, suggesting that Gd-IgA1 produced by cultured GMCs could agglomerate to form poly-Gd-IgA1. Gd-IgA1 produced by cultured GMCs presented at approximately 67 kDa in reduced conditions, while Gd-IgA1 in the serum from IgAN patients was located at 55 kDa (**Figure 4a**). The absence of a band in the MCM eliminates the possibility of Gd-IgA1 in the culture medium. Different molecular weights of cultured GMCs compared with the Gd-IgA1 from the serum of IgAN patients might be attributed to different glycosylation of Gd-IgA1. Immunofluorescence demonstrated the expression of Gd-IgA1 and C1GALT1 in HRMC and C2M12 (**Figure 4b**), suggesting that IgA in GMCs could be transformed into Gd-IgA1 in a similar mechanism as in plasma cells. Mean immunofluorescent intensity of the Gd-IgA1 staining in C2M12 was significantly lower after *IGHA1* knockdown by siRNA-2 transfection (**Figure 4c**), which further confirmed the expression of Gd-IgA1 in GMCs.

### SEB stimulated Gd-IgA1 overproduction and inflammation response in HRMCs through the TLR4 signaling pathway

**Figure 5:**
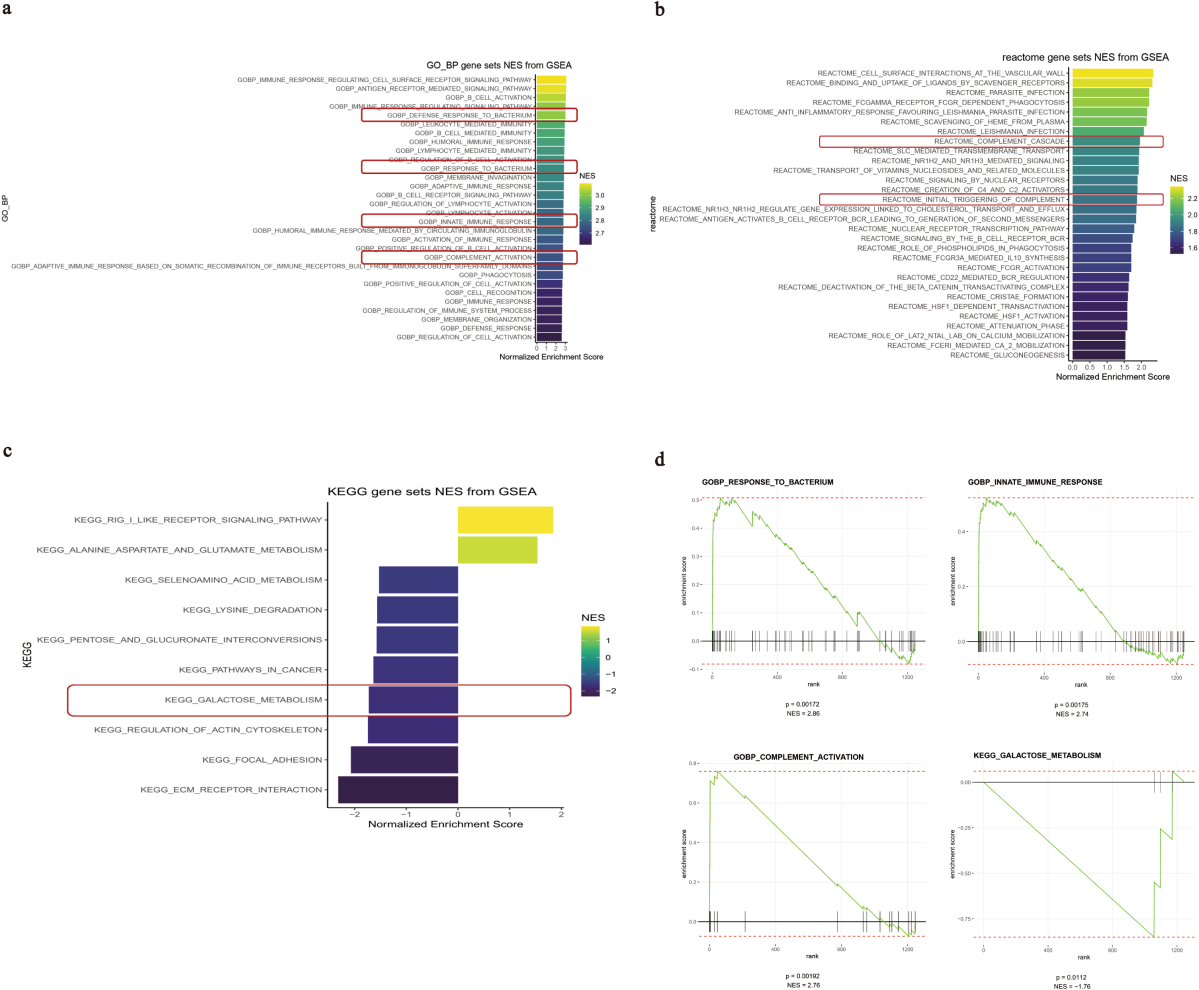
GSEA results of the *IGHA1*-associated biological functions and pathways in GMCs. **(a–c)** Top 20 functions or pathways with the highest NES associated with *IGHA1* expression based on different reference genomes. **(d)** GSEA plots of the *IGHA1*-associated functions in GMCs. GSEA, gene set enrichment analysis; *IGHA*, immunoglobulin heavy constant alpha; GMC, glomerular mesangial cell; NES, nominal enrichment score.

The top 20 biological functions or pathways enriched by GSEA with the highest nominal enrichment score (NES) significantly associated with *IGHA1* expression in GMCs are listed in **Figures 5a–c**. *IGHA1* in GMCs was positively associated with response to bacterium, innate immune response, and complement activation and was negatively associated with galactose metabolism (**Figure 5d**), suggesting that bacterium infection-induced innate immune response might be responsible for the IgA transformation into Gd-IgA1, and Gd-IgA1 might trigger the subsequent complement activation in GMCs.

**Figure 6:**
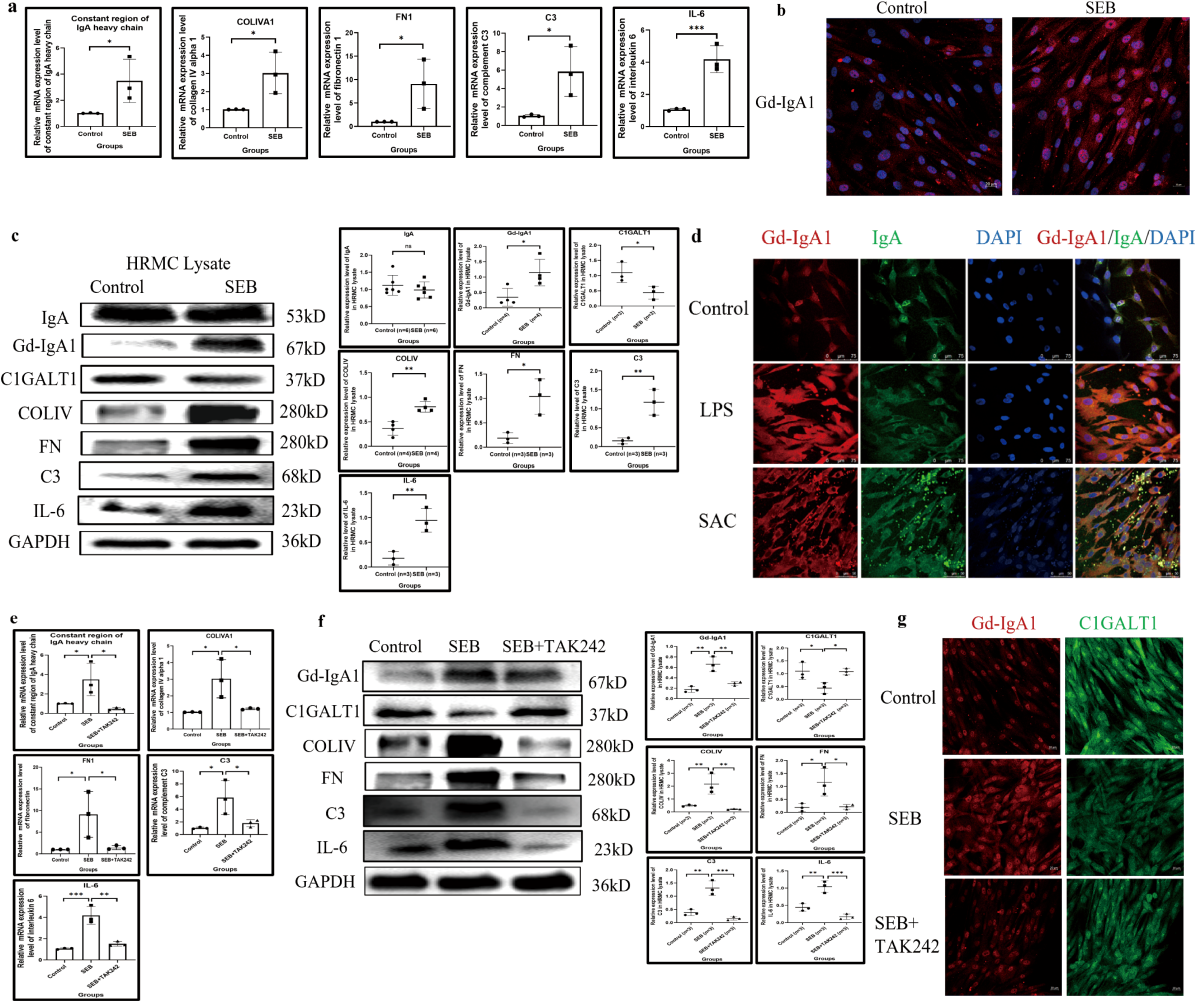
SEB induces Gd-IgA1 overproduction and inflammatory response in HRMC through TLR4. **(a)** The transcription of *IGHA1*, *COLIVA1*, *FN1*, *C3*, and *IL-6* was significantly enhanced after SEB stimulation (*n* = 3 in each group; *P < 0.05, **P < 0.01, ***P < 0.001). **(b)** Increased intracellular level of Gd-IgA1 in HRMC after SEB stimulation demonstrated by immunofluorescence. **(c)** SEB induced the increased expression of Gd-IgA1 rather than IgA in the intracellular compartment, with elevation of COLIV, FN, C3, and IL-6 production (*n* ≥ 3 per group; *P < 0.05, **P < 0.01, ***P < 0.001). **(d)** Gd-IgA1 could also be upregulated by another two bacterial stimulants in HRMC demonstrated by immunofluorescence. **(e)** The effect of SEB on enhancing the transcription of *IGHA1*, *COLIVA1*, *FN1*, *C3*, and *IL-6* in HRMC could be significantly blocked by the TLR4 antagonist (TAK242) (*n* = 3 per group; *P < 0.05, **P < 0.01, ***P < 0.001). **(f, g)** Gd-IgA1 upregulation with C1GALT1 downregulation and elevated expression of COLIV, FN, C3, and IL-6 in HRMC after SEB stimulation could be partially reversed by the TLR4 antagonist (TAK242) (*n* = 3 per group; *P < 0.05, **P < 0.01, ***P < 0.001). SEB, *Staphylococcus aureus* enterotoxin B; Gd-IgA1, galactose-deficient immunoglobulin A1; HRMC, human renal mesangial cell; TLR4, toll-like receptor 4; *IGHA1*, immunoglobulin heavy constant alpha 1; COLIVA1, collagen type IV alpha 1; FN1, fibronectin 1; C3, complement C3; IL-6, interleukin 6; IgA, immunoglobulin A; ns, not significant.

*In vitro*, SEB could significantly upregulate the transcription of *IGHA1* after 24 h of stimulation (**Figure 6a**) and induced overexpression of Gd-IgA1 in the intracellular compartment in HRMC after 48 h compared with the control group, while the IgA expression level remained relatively constant (**Figures 6b, c**). In addition to SEB, LPS and SAC could also induce Gd-IgA1 overproduction in HRMC (**Figure 6d**).

Accompanied by Gd-IgA1 upregulation in HRMC after SEB stimulation, C1GALT1 was significantly downregulated, and transcription and expression of COLIV, FN, C3, and IL-6 were significantly elevated (**Figures 6a, c**). TLRs, important members of the innate immune response, were constitutively expressed in HRMCs (**Supplementary Figure S3**), and TLR4 appeared to have the highest expression level. TAK242 (an antagonist of TLR4) could block the elevated transcription of *IGHA1*, and the expression of Gd-IgA1 relieved the abnormal expression of COLIV, FN, C3, IL-6, and C1GALT1 induced by SEB (**Figures 6e–g**).

### Gd-IgA1 deficiency relieved the inflammation response in cultured GMCs

**Figure 7:**
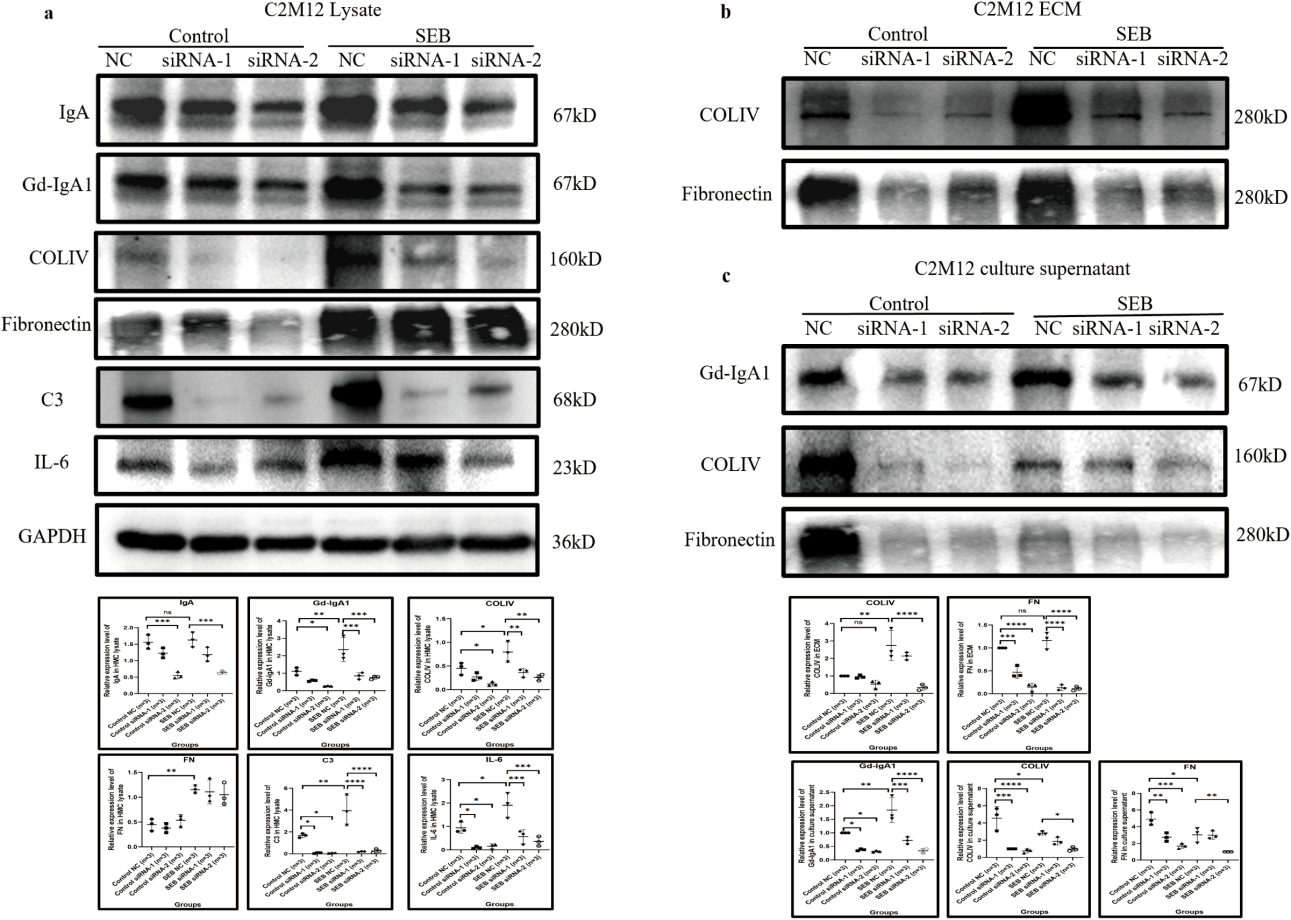
IgA knockdown by siRNA relieved C2M12 inflammation response. **(a)** IgA knockdown in C2M12 significantly reduced the production of Gd-IgA1 and COLIV, C3, and IL-6, while the FN expression remained constant (*n* = 3 per group; *P < 0.05, **P < 0.01, ***P < 0.001, ****P < 0.0001). **(b, c)** IgA and Gd-IgA1 knockdown significantly reduced the deposition of COLIV and FN in the extracellular region **(b)** and their release into the culture supernatant **(c)** (*n* = 3 per group; *P < 0.05, **P < 0.01, ***P < 0.001, ****P < 0.0001). IgA, immunoglobulin A; siRNA, small interfering ribonucleic acid; Gd-IgA1, galactose-deficient immunoglobulin A1; COLIV, collagen type IV; C3, complement C3; IL-6, interleukin 6; FN, fibronectin; ns, not significant.

*IGHA1* knockdown in C2M12 was used to investigate the pro-inflammatory effect of Gd-IgA1. siRNA-2 significantly knocked down the expression of IgA and Gd-IgA1 in C2M12 after 48 h of transfection in the condition of serum-free culture with or without SEB stimulation (**Figures 7a, c**). Gd-IgA1 deficiency reduced the production of COLIV, C3, and IL-6 in the cell lysate (**Figure 7a**) and reduced the deposition of COLIV and FN in the extracellular compartment and their release into the supernatant (**Figures 7b, c**).

### Development of IgAN in a B-cell-deficient condition and conditional knockout of *IGHA* in GMC in the IgAN mouse model

We first established the IgAN mouse model in μMT mice to exclude the effect of B-lymphocyte-originated IgA on IgAN formation, after which we managed to observe the causality between GMC-expressed IgA and the formation of IgAN in mice by conditionally knocking out *IGHA* expression in GMCs.

**Figure 8:**
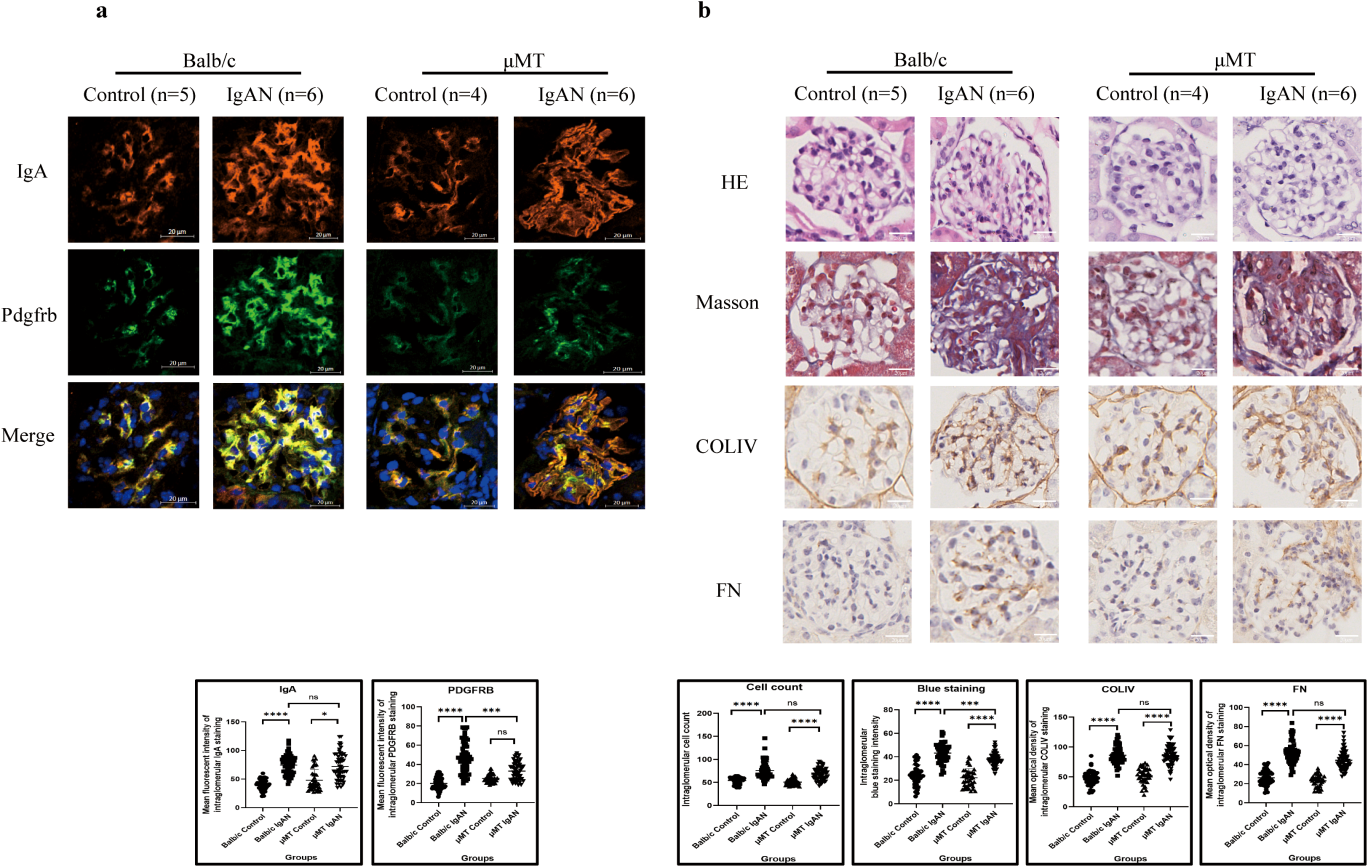
IgAN model establishment in μMT mice presented a similar pathological pattern to that in Balb/c mice (*P < 0.05, **P < 0.01, ***P < 0.001, ****P < 0.0001). **(a)** Immunofluorescence demonstrated intensified IgA staining after IgAN establishment in both μMT and Balb/c mice, while significantly excessive Pdgfrb expression was only observed in Balb/c mice. **(b)** Both μMT and Balb/c mice presented increased intraglomerular cell count and ECM expansion after IgAN establishment. IgAN, immunoglobulin A nephropathy; IgA, immunoglobulin A; Pdgfrb, platelet-derived growth factor receptor beta; ECM, extracellular matrix; HE, hematoxylin and eosin; COLIV, collagen type IV; FN, fibronectin; ns, not significant.

Similar to Balb/c mice, mesangial IgA staining was significantly enhanced after IgAN establishment (**Figure 8a**) in μMT mice, accompanied by increased inflammatory cell infiltration into glomeruli (increased intraglomerular cell count in HE staining) and ECM expansion (**Figure 8b**), while mesangial proliferation in μMT mice was not significant after model construction (**Figure 8a**).

PCR confirmed the *IGHA* homozygous genotype, and mice were classified into the *FOXD1* cre+ group (cKO) and the *FOXD1* cre− group (WT) (**Supplementary Figure S4a**). Mean fluorescence intensity of intraglomerular IgA staining in cKO mice was significantly lower than that in WT mice, confirming the *IGHA* knockout efficiency in GMC, in which platelet-derived growth factor receptor beta (PDGFRB) served as a marker of GMCs (**Supplementary Figure S4b**). As we used the *FOXD1* cre system for conditional knockout, IgA expression in CD19^+^ cells in the spleen and the serum IgA level remained constant between the WT and cKO groups (**Supplementary Figure S4c**).

**Figure 9:**
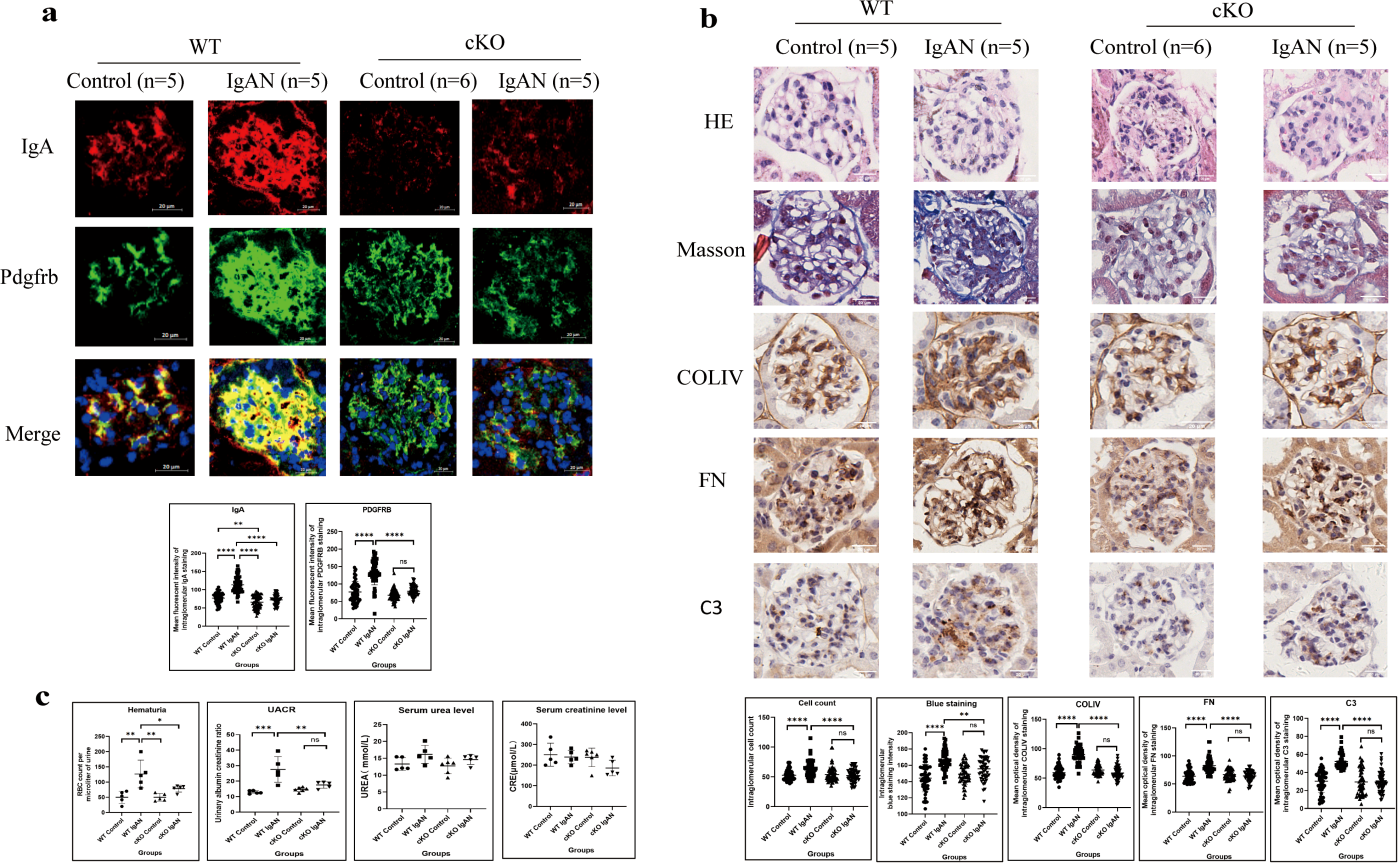
IgA expressed by GMCs induces intraglomerular inflammatory response in IgAN in mice (*P < 0.05, ***P < 0.001, ****P < 0.0001). **(a)** Immunofluorescence demonstrated intensified IgA and Pdgfrb staining in the IgAN group of the WT mice compared to the control group; conditional IgA knockout in GMCs reduced the IgA and Pdgfrb expression in the GMCs. **(b)** Histological analysis demonstrated increased cell count (HE staining), enhanced collagen deposition (Masson’s trichome staining), and increased deposition of COLIV, FN, and C3 (IHC) in the glomeruli of the IgAN group compared with the control group in WT mice, and conditional *IGHA* knockout in GMC significantly relieved intraglomerular hypercellularity and ECM and C3 deposition. **(c)** Urinary RBC count and UACR in the IgAN group were significantly higher than those in the control group in WT mice; hematuria and proteinuria were relieved significantly after conditional *IGHA* knockout, while the difference between serum urea and creatinine levels among the groups was insignificant. GMC, glomerular mesangial cell; IgAN, immunoglobulin A nephropathy; IgA, immunoglobulin A; Pdgfrb, platelet-derived growth factor receptor beta; WT, wild type; HE, hematoxylin and eosin; COLIV, collagen type IV; FN, fibronectin; C3, complement C3; IHC, immunohistochemistry; ECM, extracellular matrix; RBC, red blood cell; UACR, urinary albumin–creatinine ratio; CRE, creatinine; ns, not significant.

In WT mice, intensified IgA in glomerular mesangium was observed in the IgAN group compared with the control group, with significant mesangial hypercellularity proven by PDGFRB staining and HE staining (**Figures 9a, b**). Masson’s trichome staining revealed more collagen deposition, and IHC demonstrated more deposition of COLIV, FN, and C3 in the mesangium in the IgAN group compared with the control group (**Figure 9b**). Significantly deteriorated hematuria and enhanced UACR were also found in the IgAN group (**Figure 9c**). Stronger IgA expression in the mesangium and significant hematuria and proteinuria with enhanced intraglomerular inflammation response indicated the successful construction of the IgAN mouse model.

Compared with the WT IgAN model, less IgA expression in the mesangium was detected in cKO IgAN mice (**Figure 9a**). GMC proliferation indicated by PDGFRB staining (**Figure 9a**) and HE staining (**Figure 9b**) and extracellular matrix deposition by Masson’s trichome staining and IHC (**Figure 9b**) were all alleviated in the cKO group compared with the WT group. Moreover, deficient IgA expression in GMCs also led to reduced C3 deposition in the mesangium (**Figure 9b**) and alleviation of hematuria and proteinuria (**Figure 9c**) in the cKO IgAN model. However, the kidney function of mice in the IgAN group did not change significantly which might be attributed to the early stage of the disease (**Figure 9c**). To sum up, these results indicated that IgA expression in GMCs was responsible for inducing intraglomerular inflammation and alleviating the hematuria and proteinuria in IgAN in mice.

## Discussion

In this study, we reconfirmed the expression of IgA and Gd-IgA1 in GMCs and preliminarily revealed that Gd-IgA1 overproduction in GMCs induced by SEB through the TLR4 pathway may play a role in the pathogenesis of IgAN.

We previously found that GMC could express IgA with IgA1 being dominant as in IgAN, and SEB could stimulate the production of IgA (17, 18). In this study, transcription of the V_H_DJ_H_ gene rearrangement of *IGHA* was detected in single GMCs isolated from renal biopsies of IgAN patients. The transcription of *IGHA1* in GMCs was also confirmed in a public single-cell RNA-sequencing database. Liu (23, 24) reported the transcription of *IGHA* in mouse GMCs. Zheng (25) discovered the upregulation of the J chain, the necessary component for IgA polymerization, in GMCs in IgAN patients, also suggesting that there might be IgA synthesis and polymerization in mesangial cells.

IgA “deposition” in normal renal tissues was assessed as a preliminary attempt to find out if GMCs in human renal tissues can express IgA. IgA was stained convincingly, although the staining was light and sparse in normal tissue compared to the thick and dense staining in IgAN renal biopsies. *In situ* expression of *IGHA1* by GMCs was then confirmed by FISH. IgA expression in glomerular mesangium was also detected in μMT mice, which lack B cells and serum IgA (26). The IgA expression in μMT mice was clear and precise, even with a higher intensity than that in wild-type mice.

As Gd-IgA1 overproduction due to C1GALT1 downregulation in response to microbial stimulation is the most important factor that initiates the pathogenesis in IgAN, the serine/threonine-rich region in the *IGHA1* transcripts and Gd-IgA1 and C1GALT1 expression were detected in cultured GMCs, which suggested that IgA1 in GMCs could be transformed into Gd-IgA1 in a similar mechanism as in plasma cells (6).

Bacterial infection is a common trigger for IgAN development and progression. Infectious stimulants were utilized to induce overexpression of Gd-IgA1 in CMCs. TLRs were all found to be expressed in GMCs, with TLR4 as the dominant one in our study, suggesting that GMCs carry the capability of inducing an inflammatory response in bacterial infection. The effect of TLRs on accelerating IgAN progression has been studied by several studies. Upregulation of TLR4 in circulating mononuclear cells was found to be associated with proteinuria and hematuria in IgAN patients (32), and TLR4 in intestinal mucosal tissue and renal tissues might accelerate IgAN progression through the TLR4–MyD88–NF-κB pathway (33, 34). Activation of TLR9 and TLR7 could also enhance kidney injury in IgAN by inducing Gd-IgA1 overproduction through the APRIL and IL-6 pathways (35, 36). Interestingly, single-nucleotide polymorphisms of TLR9 were associated with IgAN progression (37).

IgA knockdown by siRNA was performed to investigate the pro-inflammatory effect of Gd-IgA1. Reduced Gd-IgA1 production and release by IgA knockdown led to the reduced production of COLIV, C3, and IL-6 in the cell lysate and relieved COLIV and FN deposition and release induced by SEB, indicating that Gd-IgA1 overexpression under bacterial stimulation could enhance the inflammation response in GMCs.

To exclude the effect of B-cell-originated IgA, we compared the IgAN model in μMT mice to Balb/c mice and discovered that IgAN in μMT mice developed a similar pattern of IgA deposition in the mesangium and ECM expansion in Balb/c mice. Then, Foxd1 cell population-specific *IGHA*-deficient mice were developed by crossing *IGHA*^flox/flox^ mice and Foxd1^GC^ mice to observe the causality between the GMC-expressed IgA and the formation of IgAN in mice, as it has been reported that GMCs were one of the major targets in Foxd1 lineage-specific gene deletion (38). Compared with the control group, the IgAN model in WT mice displayed stronger IgA expression, mesangial hypercellularity, and more C3 and ECM deposition in the mesangium with enhanced hematuria and proteinuria. Compared with the WT IgAN model, less IgA expression, reduced GMC proliferation, and extracellular matrix deposition in the mesangium with alleviated hematuria and proteinuria were detected in the cKO IgAN model, indicating that IgA deficiency in GMCs could relieve IgAN.

The limitations of this study should be noted. Firstly, we only detected a small amount of GMCs with the *IGHA* V_H_DJ_H_ gene rearrangement and transcription through nested PCR combined with Sanger sequencing, as high-throughput RNA sequencing hardly detected the V_H_DJ_H_ gene rearrangement in non-B cells. Secondly, we still cannot distinguish Gd-IgA1 generated by GMCs from Gd-IgA1 generated by plasma cells. Distinguishing Gd-IgA1 generated by GMCs from that by lymphocytes might help improve our understanding of IgAN.

## Conclusion

In this study, we reconfirmed the expression of IgA in GMC and preliminarily revealed that Gd-IgA1 induced by SEB through TLR4 in human GMC may play a role in the pathogenesis of IgAN.

## Data Availability

The original contributions presented in the study are included in the article/**Supplementary Material**. Further inquiries can be directed to the corresponding author/s.
