# Supplementary material for: IgA expressed by glomerular mesangial cells is involved in the pathogenesis of IgA nephropathy
Source: Front Immunol. 2025 Nov 25;16:1638818. doi: 10.3389/fimmu.2025.1638818 (PMC12685827; doi:10.3389/fimmu.2025.1638818)
Supplement: Supplementary file 1 [file Table1.docx]

| siRNA | Direction | Sequence (5’-3’) |
| --- | --- | --- |
| siRNA-1 | Forward | GCUCUUAGGUUCAGAAGCGTT |
|  | Reverse | CGCUUCUGAACCUAAGAGCTT |
| siRNA-2 | Forward | GCCUUCACACAGAAGACCATT |
|  | Reverse | UGGUCUUCUGUGUGAAGGCTT |

**Supplementary Table 1**. Sequences of siRNAs used in this study.

SiRNA: small interfering RNA.

**Supplementary Table 2**. Reaction conditions of polymerase chain reaction used in this study.

| Species/  Method | Gene name | Initial denaturation (˚C/min) | Denaturation (˚C/sec) | Annealing (˚C/sec) | Extension (˚C/sec) | Cycle number | Extension (˚C/min) |
| --- | --- | --- | --- | --- | --- | --- | --- |
| Human/  RT-qPCR | *C3* | 95/2 | 95/5 | 60/10 | - | 35 | - |
|  | *IL6* |  |  |  |  |  |  |
|  | *COLIVA1* |  |  |  |  |  |  |
|  | *FN1* |  |  |  |  |  |  |
|  | *IGHA1* |  |  |  |  |  |  |
|  | *ACTB* |  |  |  |  |  |  |
| Human/  standard PCR | *ITGA8* | 95/5 | 95/30 | 58/30 | 72/30 | 30 | 72/10 |
|  | *ACTA2* |  |  |  |  |  |  |
|  | *GAPDH* |  |  |  |  |  |  |
| Mouse/  standard PCR | *FOXD1* | 94/2 | 94/15 | 60/15 | 72/10 | 28 | 72/2 |
|  | *IGHA* | 95/5 | 95/30 | 65/30 | 72/30 | 30 | 72/10 |

RT-qPCR: Real Time-quantitative Polymerase Chain Reaction; PCR: Polymerase Chain Reaction

**Supplementary Table 3**. Sequences of polymerase chain reaction primers used in this study.

| Species | Gene name | Direction | Primer sequence 5'‑3' | Product length (bp) |
| --- | --- | --- | --- | --- |
| Human | *C3* | Forward | CTGTCCACGACTTCCCAGG | 131 |
|  |  | Reverse | CCCCTTTTCTGACTTGAACTCC |  |
|  | *IL6* | Forward | ACTCACCTCTTCAGAACGAATTG | 149 |
|  |  | Reverse | CCATCTTTGGAAGGTTCAGGTTG |  |
|  | *COLIVA1* | Forward | ATGTCAATGGCACCCATCAC | 382 |
|  |  | Reverse | CTTCAAGGTGGACGGCGTAG |  |
|  | *CH1-H-CH2* | Forward | ACATGCCACGTGAAGCACTA | 398 |
|  |  | Reverse | GTTAGCGGGGTCTTGGACTC |  |
|  | *FN1* | Forward | CGGTGGCTGTCAGTCAAAG | 130 |
|  |  | Reverse | AAACCTCGGCTTCCTCCATAA |  |
|  | *IGHA1* | Forward | ACCATGCAGGAGAAGGTTGTC | 340 |
|  |  | Reverse | TCACTTGCACTGCTGCCTAC |  |
|  | *ACTB* | Forward | AGAGCTATGAGCTGCCTGAC | 121 |
|  |  | Reverse | AATTGAATGTAGTTTCATGGATG |  |
|  | *ITGA8* | Forward | GCCTATGCCGAGTTCTCTCC | 297 |
|  |  | Reverse | CCCAGTAAACTCCCCAGCAG |  |
|  | *ACTA2* | Forward | CAAGTCCTCCAGCGTTCTGA | 173 |
|  |  | Reverse | TCCCGGGGATAGGCAAAGT |  |
| Mouse | *FOXD1* | Common | TCTGGTCCAAGAATCCGAAG | WT: 237  Mut: 450 |
|  |  | WT Forward | CTCCTCCGTGTCCTCGTC |  |
|  |  | Mutant Forward | GGGAGGATTGGGAAGACAAT |  |
|  | *IGHA* | Forward | TGCCTGCATGGCATGGACAGA | WT: 222  Mut: 346 |
|  |  | Reverse | GCCAGCACGGGGTAGGAGTG |  |

WT: Wild Type; Mut: Mutant.


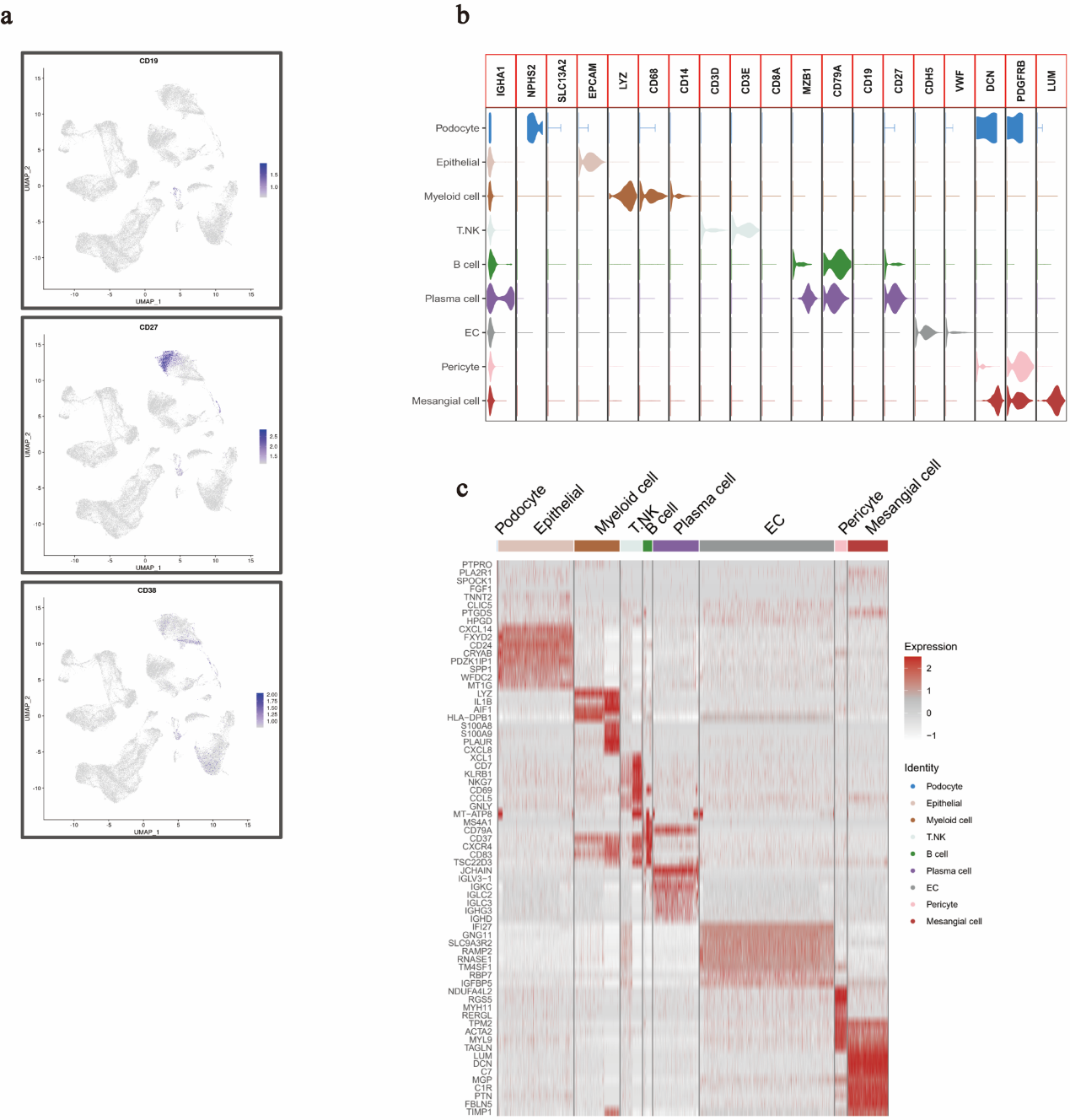


**Supplementary Figure 1**. Re-analysis of the kidney scRNA-seq data from the public database. **a** UMAP plots of the *CD19*, *CD27* and *CD38* expressing B cells and plasma cells. **b** Re-clustering of the IgHA1 expressing cells based on known cell type markers. **c** Heatmap of the top 8 differentially expressed genes in IgHA1 expressing cell types.


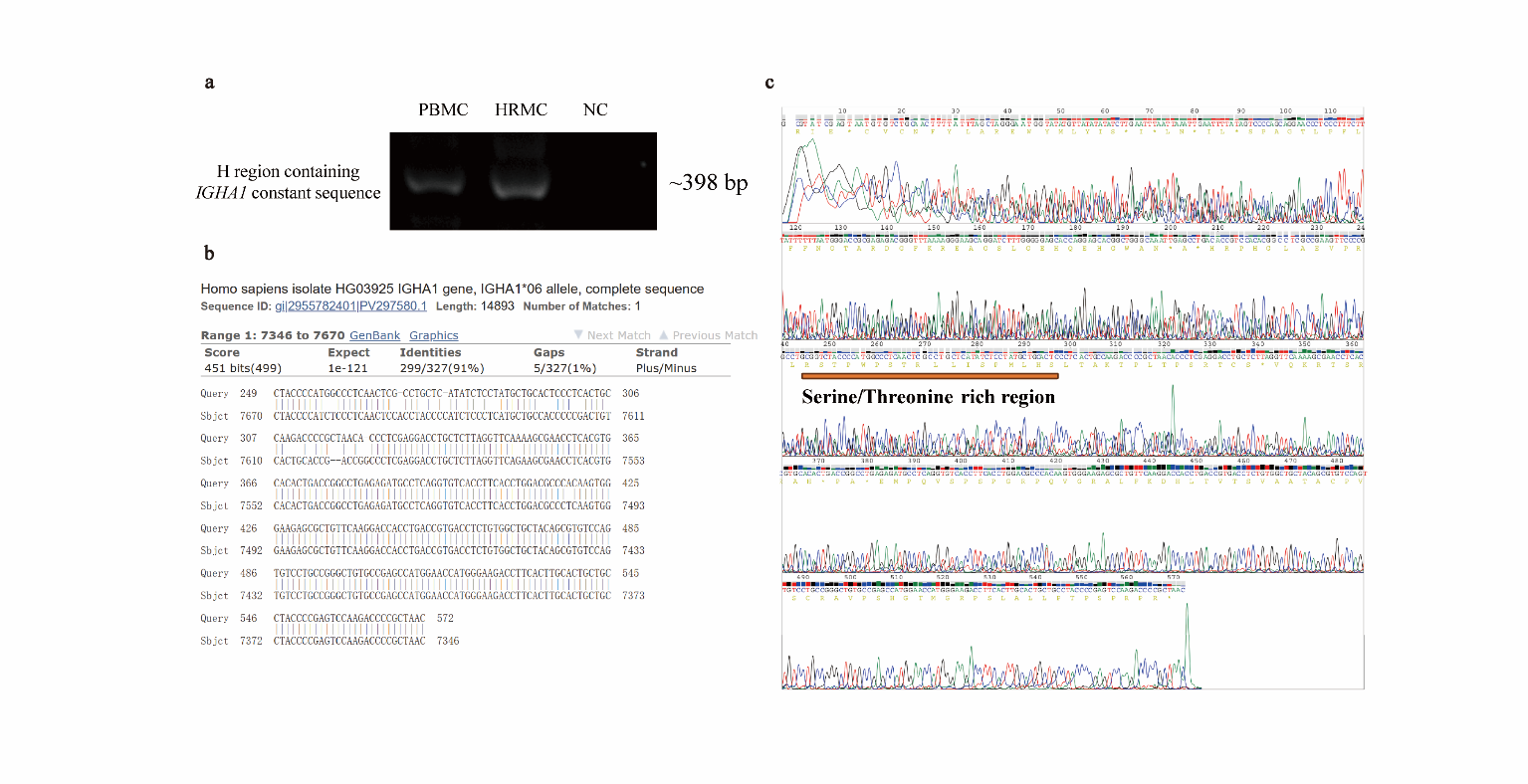


**Supplementary Figure 2**. Detection of serine/threonine rich region in *IGHA1* transcripts produced by HRMC. **a** The product length of H region containing IgHA1 constant sequence (*CH1-H-CH2*) produced by HRMC was similar with that produced by PBMC. **b** Alignment of the PCR product sequence from HRMC with the *IGHA1* mRNA sequence from the NCBI database confirmed transcription of *IGHA1* constant region in HRMC. **c** The serine/threonine rich region of the IgHA1 transcript from HRMC located at the same region as that in the B lymphocytes but with a different amino acid sequence.

IGHA: [Immunoglobulin Heavy constant Alpha](https://www.ncbi.nlm.nih.gov/gene/238447); HRMC: Human Renal Mesangial Cell


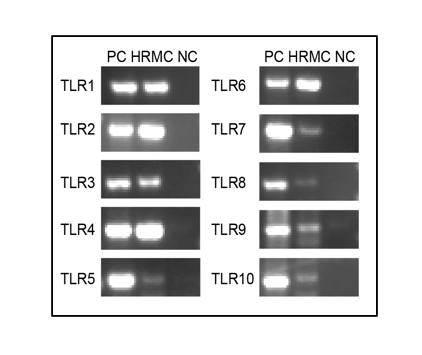


TLR: Toll-Like Receptors; PC: Positive Control; HRMC: Human Renal Mesangial Cell; NC: Negative Control

**Supplementary Figure 3**. Transcription of Toll-like Receptors in HRMCs by PCR. Peripheral blood monocytes and water serve as positive control and negative control for TLRs transcription, respectively.


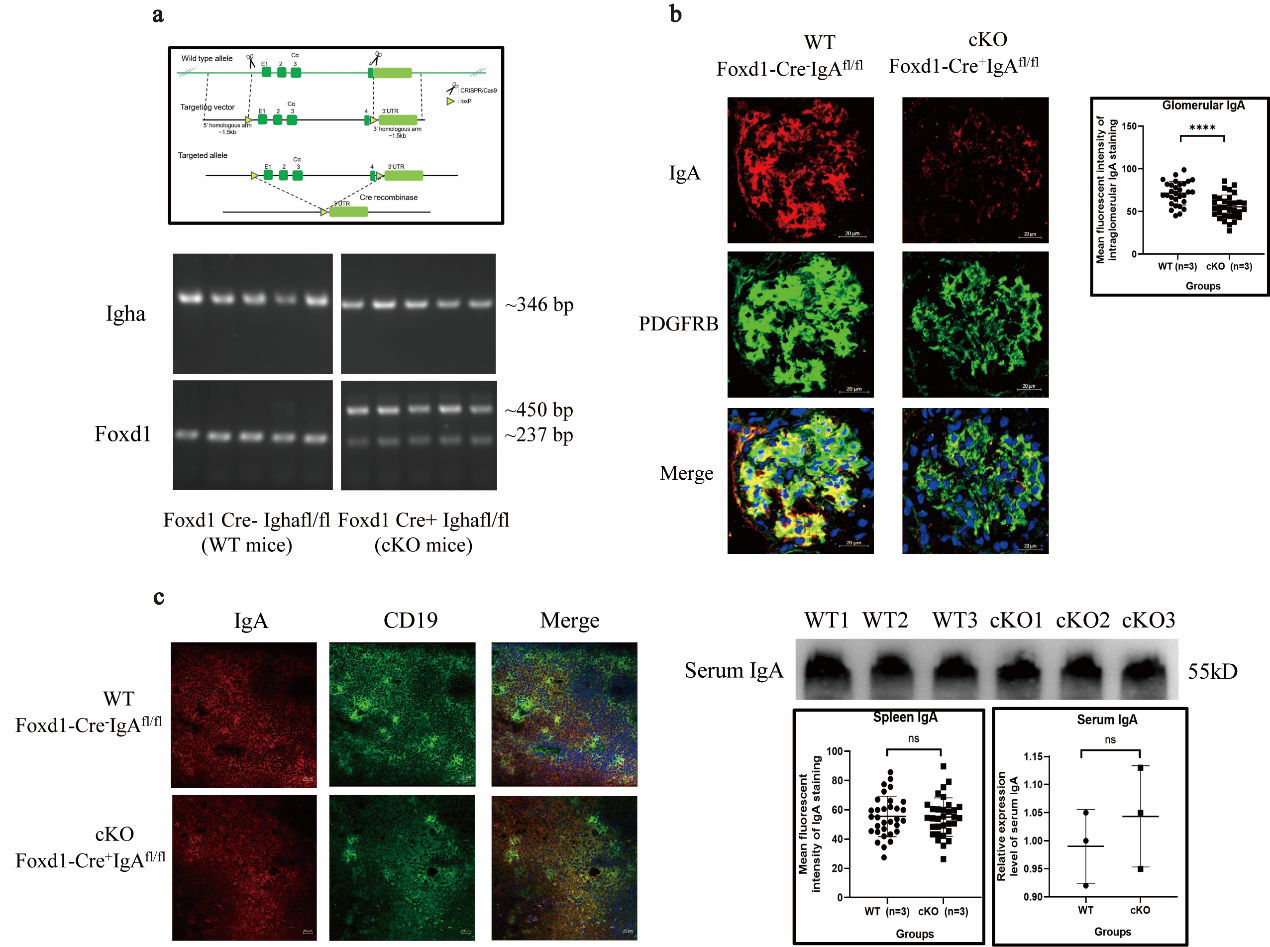


WT: Wild Type; Cko: conditional KnockOut; CD19: B lymphocyte marker.

**Supplementary Figure 4**. Knockout efficiency and specificity of the gene editing mice. **a** Confirmation of the Igha homozygous genotype and classification of the mice into Foxd1 cre+ group (cKO) and Foxd1 cre- group (WT) by PCR. **b** The IgA expression in GMCs was significantly lower in the cKO mice compared to the WT mice (n=3 per group). **c** Constant IgA expression level in spleen and serum between WT and cKO mice confirmed that conditional knockout of IgHA in mesangial cells did not affect the expression of IgA in B lymphocytes (n=3 per group).
